# Greening clubs- the role of sport in Ireland's approach to local climate action

**DOI:** 10.3389/fspor.2026.1694471

**Published:** 2026-05-20

**Authors:** Evan Boyle

**Affiliations:** The Sustainability Institute, University College Cork, Cork, Ireland

**Keywords:** climate action, GAA, regime-based transition intermediaries, social theory, sustainability, sport

## Abstract

Climate change highlights the importance of an all of society response including bottom-up and community led action. Increasingly, sports clubs have a role to play in engendering local level sustainability. In the Irish context, the Gaelic Athletic Association (GAA) is central to sporting life. It was established in 1884 with the aim of building a sense of Irish identity and community, with which it is now entwined. It promotes the indigenous sports of Ireland, namely handball, camogie, hurling, and Gaelic football. This research outlines the history of the GAA, its role as a local intermediary for climate action, the first two phases of the ‘Green Club’ community sustainability programme, and plans for government expansion. We develop an intrinsic case study, using exploratory interviews and reliance on secondary sources, with a focus on document analysis in relation to the Green Club programme, to answer the question “how do local sports clubs implement and benefit from a nationally coordinated sustainability programme?”. We provide reflections on competitive sporting organisations as a form of intermediary between national objectives and local action. This work contributes to the literature by presenting the case as an example of potential mechanisms for enabling climate action through sports clubs at the collective level.

## Introduction

The depth and range of transformative change needed in responding to the climate crisis necessitates a whole of society approach to climate action. Within this, such a transformation cannot be led by state actors alone but instead must enable collaboration across society. Local sports organisations can prove fruitful avenues to facilitate bottom-up climate action. Their reach within communities coupled with their established structures, purpose, membership, and history provide strong societal infrastructure to enable action.

**Figure 1:**
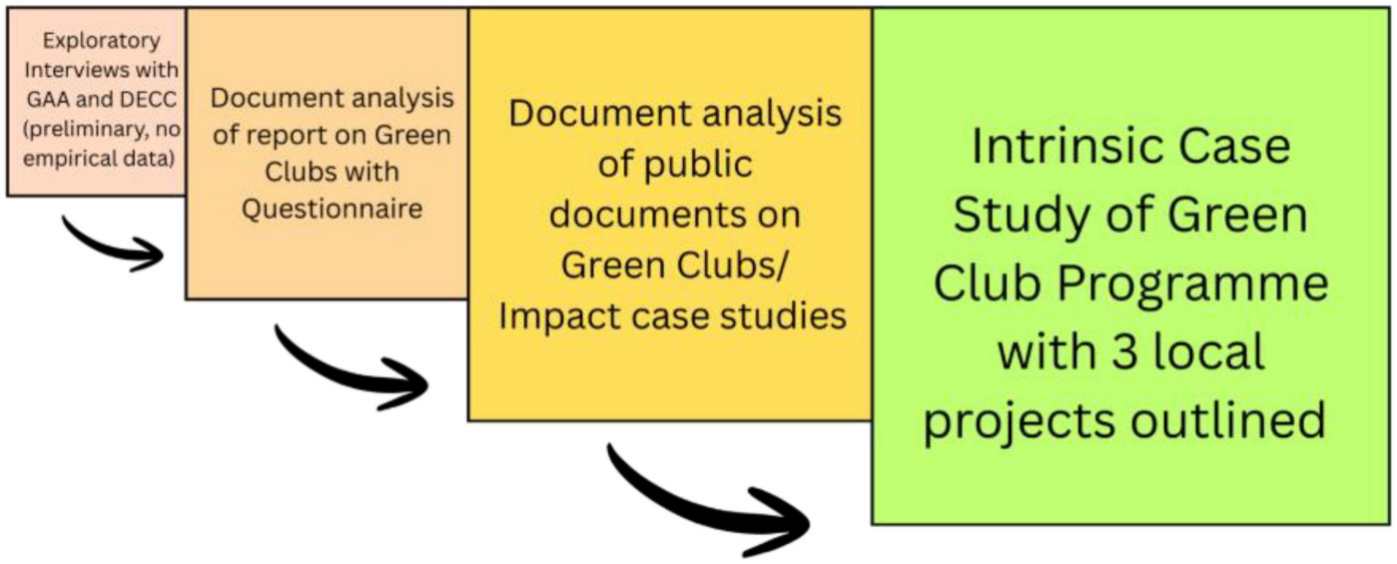
Three phased methodological approach to intrinsic case study formation using exploratory interviews and document analysis to create intrinsic case study of green club programme with 3 local projects outlined.

### The civilising process: sport, sustainability, and the GAA

As a ritual process, situated between play and work, sport has long since been theorised as having a crucial role in building social solidarity, differentiating social roles, and negotiating power dynamics. For Bourdieu, sport is shaped by social class and habitus, the way in which individuals perceive the world around them informed by habits, skills, and social environment. For Gramsci, dominant ideologies can be both reproduced and contested through sport. While for Baudrillard, sport is a hyperreal simulation mediated through mass communication channels and the enacting of large-scale sporting events. While sport has been of critical consideration within the sociological canon from the middle of the 20th Century, a deeper theoretical investigation is outside the scope of our purpose here [see: (1)]. However, the work of both Elias and Dunning (2), in weaving the importance of sport through the *civilising process,* is worthy of regard in relation to our own investigation. Elias, as an increasingly recognised key figure in 20th century sociology, was perhaps the first major sociologist to give serious consideration to the importance of sport in relation to understanding the totality of social life.

Within our context, the history of the GAA can be usefully understood through the contextual lens developed by Dunning and Elias in *Quest for Excitement in Sport* (2), and applied to the GAA (3, 4). Both Gaelic games, hurling and Gaelic football share some similarities. They are both played on the same field, and both on the ground and in the air. The goalposts are a H structure similar to rugby, with a goalkeeper and netting on the lower portion. A score with the ball above the crossbar is 1 point while below it, a goal, is three. Both games are fifteen players per team, played over two 35-minute halves. In hurling, a wooden stick with a broad base, a hurl, is used to compete over a small ball, a sliotar. In Gaelic football, the ball is kicked, fisted and caught (5). In analysing the civilising process as applied to both hurling (3) and Gaelic football (4), the evident “sportization” of both has resulted in increased emotional restraint both of the spectators and the players in parallel with similar such developments within Irish society due to increased interdependencies between the citizenry. This civilising process, or a form of rationalization in a Weberian sense, can still be seen as ongoing. For example, in the case of hurling the introduction of mandatory helmet wearing was implemented as recently as 2010 across the GAA.

Gaelic games have for centuries been linked to the ethnic identity of Ireland and have existed in parallel to political and cultural currents. Hurling has deep ancient roots going back more than 2,000 years and traced out through the mythic sagas of pagan Ireland as seen through Cuchulainn and the Tuatha de Danaan. Throughout British occupation, the game was a form of political resistance and was deemed an unsuitable activity by the colonial administration. Throughout the occupation there are numerous examples of direct action to prevent the playing of hurling such as the 1527 ‘Statute of Galway’ which ordered that hurling not take place and in 1695 ‘Sunday Observance Act’ which stated that “no person or persons whatsoever, shall play of exercise any hurling” [(6), P.481]. Reference to Gaelic football emerged in the 1600s, although a precursory form was most likely practiced throughout the Middle Ages. Similar attempts towards banning football were also set out through the Sunday Observance Act (1695).

Established in 1884 with the stated aim of protecting against the declining popularity of the indigenous games, the GAA has for 125 years played a critical role in the formulation of Irish identity from the home rule movement to independence from Britain up to the modern globalised Irish state. The depth of academic enquiry into the GAA and its influence on Irish society and identity is presented across a broad range of disciplines including sociology, psychology, economics, and history [See: (7–8)]. Rather than seeking to provide a holistic overview of this literature, here we consider the broader context of the organisation and its potential function for non-sport related engagement. The GAA throughout the 20th Century was entwinned with a burgeoning Irish nationalism. On reflection of the GAA's centenary celebrations, Ryan notes that the organisation; “has its origins in the intensifying nationalist atmosphere of late nineteenth century Ireland and was indeed avowedly nationalist from the beginning” [(9); 755]. Several of the GAAs early laws aligned with this sentiment. Rule 27, introduced in 1905 and amended in 1971, forbid any members of the GAA from playing or attending British-origin sports. Rule 42, beginning in the early 20th Century and relaxed since 2005, banned the playing of non-Gaelic games in GAA owned stadiums. The role of the GAA in seeking to exert influence on shaping societal norms is still seen today. In a recent case which garnered international attention and ridicule, the organisation sought to continue with the imposition of skort (running skirt) wearing on female athletes (10). This highlights the continually negotiated influence of the GAA on Irish society, and the different lines of acceptance which are threaded.

The relationship between sport and society in Ireland has changed many times throughout the 20th century [(11), P. 13]. Throughout the early 1900s the GAA oscillated between decentralising and centralising tendencies, culminating ultimately in more integration and centralisation as the network of local factions became longer and more weighted (5). While in modern times, the GAA Official Guide still aligned with a sense of nationalism and the consolidation of Irish identity both at home and abroad, the professionalisation of the organisation up to and within the 21st Century (whilst still being defined by amateurism at the elite level) ties itself to universal operational considerations evident in sporting organisations across the globe (12). Within this context, the GAA can be seen as a vehicle through which to actualise responses to societal and policy challenges in alignment with governmental objectives. In relation to public health, social exclusion, and environmental sustainability, initiatives have been developed in partnership with the organisation for implementation in communities across the country. It is seen as one of the greatest achievements of the GAA that it has established a functioning mechanism for the expression of a collective identity in Ireland.

“It took the rather crude forms of hurling and football which existed in nineteenth century Ireland and fashioned them into organized sporting contests through which the nation as a whole, and every parish and community within it, could express its feeling of rivalry and superiority in a socially approved and culturally appropriate manner. People identified with parish and county teams with an intensity never reached in any other social organization whether political, economic of even religious. In these activities, prestige was sought for the village and community as a whole, and all members, whether active players or not, gloried in the victory” [(9); 757].

The community cooperation is borne of competition and the pursuit of prestige against a nearby rival. Fierce competition mixed with strong cooperation can be seen across the local GAA club network. Liston (13), describes it as a unique blend of solidarity and rivalry (P.201). Beyond the sporting competition and solidarity which it provides, a formidable infrastructure has been established at the national level which can be leveraged for policy implementation. Both predating and providing a blueprint for the Green Club programme which we will consider, is the Healthy Club programme. Since 2013, this initiative instigates the promotion of health within and across the organisation (14). The links between the GAA and community building, inclusion, and social justice have recently been traced, with its “deeply rooted presence in Irish life, the GAA demonstrates how sport can function not merely as a site of athletic competition but as a dynamic and responsive civic institution, fostering leadership, empathy, and resilience... and building stronger, more cohesive communities” [(15), P. 130]. Coupled with this, “we-group” identities are created through competition, and rivalries are central to the GAA (4).

### The role of intermediaries in climate action

A whole of society approach to responding effectively to the scale of challenges posed by climate change requires the merging of both top-down and bottom-up approaches. The Multi-Actor Perspective (MaP) (16) acts as a framework for interpreting the relationships and interactions between actors within sustainable transitions. The MaP provides a useful heuristic device for understanding changing actor roles within transition studies (17). The MaP can analyse governance within transitions and the politics of sustainable transitions. To understand governance and politics, however, a better appreciation of actors involved in transitions is warranted. The power dynamics between different actors, acting at different scales, if properly understood, can lead to insightful findings with regards to conceptualising relationships. The MaP distinguishes between the sectors of state, market, community and third sector. Alongside this, the heuristic tool functions at a scalar level, between sectors, organisational actors and individual actors. The scalar classification functions to capture the movement of actors, whereby at different levels actors can operate within different sectors i.e., state, market, community and third sector. On from this “the MaP serves to question and problematise how these institutional logics are constructed, and then to explore the dynamic between and across different institutional logics as well as how individuals, groups and organisations act and relate within and across these logics” [(18), Pp.4–5]. By way of example, at the individual level an actor may operate as a volunteer within a community group. At an organisational level this same person may be representative of a market actor. The need to interpret the versatility of actor roles is afforded by the MaP. This framework allows for analysis of the shifting power relations within sustainability transitions (16).

A systematic exploration of the literature on actors and agency in the sustainability transition literature identifies four typologies of actors: 1- Systemic actor typology (niche, regime, landscape); 2- Institutional actor typology (state, market, civil society); 3- Governance actor typology (actors at local, regional, national and global governance level); 4- Intermediaries (19). Intermediaries have been defined as providing and distributing necessary information, mediating and service provision, connecting niche level activities with regime level institutions and diffusion of new technologies. The agency of intermediaries is defined as resource dependent, with them operating as active agents. Intermediaries, due to their very nature can operate beyond their own typology to be present within any of the other three, operating as systemic, institutional or governance actors, however, more distinct definitions of intermediary types serves to illuminate the diversity contained within the broad categorisation outlined here and the ecology of intermediaries framework discussed below (20).

Intermediaries are essential in promoting and enabling the start-up and continuation of grassroots projects from a bottom-up standpoint (21, 22). Diffusion and scaling-up of individual grassroots projects can be facilitated through intermediary support. Consequently, they play a function in communication; “as traditional boundaries between actor groups are being eroded or redefined, intermediaries would appear to play an important role in communicating across cultures of compliance (state), of competition (market) and of collaboration (civil society)” [(23), P. 1492]. The linking of these ‘cultures’ may prove fruitful and the potential for a timely transition could be framed optimistically due to the diversity of actors at a broad level engaged to govern the transition to a low- carbon socio-technical system (24).

The discussion of Kivimaa et al., on intermediation (20) has highlighted transition intermediaries operating at the widest level with reference to the transition, whereby they can be seen as linking or connecting skills and resources, or visions and demands.

The suggestion here is that different transition intermediaries have different roles but act in ‘ecologies of intermediaries’. Nevertheless, they provide a wide range of functions: including articulation of expectations, demands and visions; creating and brokering networks; exchange of knowledge and support of learning processes; innovation process management; translation between different actors, interests and contexts; capacity building, including the creation of required knowledge; institutional support; and configuration of local technological assemblages (20).

Moving beyond an understanding of the functions which intermediaries provide, we can begin to consider the spaces which they operate within. Recent work (25), uses relational space theory to suggest that the relational nature of intermediaries can overcome binary state/community interpretations. On from this, the power dynamics at play in intermediary spaces manifests both within them and beyond them. They are seen here not as neutral spaces. From this perspective, the nature of intermediary spaces is seen as dynamic, offering the potential for new spaces to emerge and challenge the status quo.

The development of an analytical perspective built upon middle actors has been established within the literature and utilised in several contexts from domestic heating to solar installation (26, 27). As an analytical devise, it represents often ignored actors within the system to help interpret their potential effectiveness in expediting the transition to a low-carbon society. The need for a framework which indicated the influences of middle actors helps to move discussion of intermediaries away from them being solely viewed as “go-betweens” of top-down and bottom-up engagement, one step removed from the process [(28), P. 109]. Intermediaries have the potential to play a multitude of roles within societal transitions (20, 29–31). This idea of a middle actor represents longevity in a way which traditional understandings of intermediaries overlooks. Intermediaries have also been investigated in terms of strength of engagement with a project and also the temporal element of this engagement, be it short, medium or long term (32). As Cronin notes, “for the GAA, a sense of identity is not created on the back of a sense of community, but is created from above” [(33), P. 100]. This is to say that the GAA, while operating as a dispersed network of locally embedded volunteer clubs, has since its establishment been heavily coordinated centrally and strategically.

The GAA has been referenced as an “alternative organisation” with a particular understanding of “management”, an emphasis on place and community, is volunteer led; operates on democratic principles; and has a remit or interest beyond purely sporting endeavours (34). Its role as a regime-based transition intermediary is clearly illustrated through its centralised control and localised impact. Below, we have sought to outline the Green Club initiative as a case study which can be usefully explored as an example of the role of sports organisations to operate in a remit beyond their primary function. In doing so they hold the potential to facilitate the emergence of grassroots action in relation to the climate action agenda. Yet, operating in this space may also present challenges and conflicts at the local level.

## Methods

To complete this study, both public and internal documentation surrounding the first and second phases of the Green Club programme was analysed. A three-part process, as outlined below, was followed. In the first instance exploratory interviews were held to understand the context in which the Green Club programme has emerged and to assess the viability of undertaking research work in this space. Two interviews were held. Firstly, with the Youth Leadership and Sustainability Manager at the GAA and secondly, a civil servant within the Department of Climate, Energy and the Environment who supported the establishment of the Green Club initiative. These scoping interviews were not recorded and instead notes were taken through the process, these act as preliminary knowledge gathering for the study rather than empirical data gathering. These initial interviews helped to provide an initial grounding about the Green Club programme and provided important preliminary knowledge. This led to the analysis of publicly available documentation on the Green Club programme. Many case studies related to different sectoral priorities are available through the GAA website. Coupled with this, a request was made for further documentation firstly in relation to the national development of support through policy for sustainability in sport and secondly, to the GAA in relation to their Green Club approach. A report on the Green Club programme was made available for analysis. The results of this case study concerning the experimentation with climate action through sport in the Irish context are supported with a wider review of the GAA and identity in Ireland, and the literature on intermediation as outlined above.

Case study research has received much criticism for lacking the ability for hypothesis testing, lack of generalisability, the potential for bias towards verification, and an over reliance on practical rather than theoretical insights. While criticisms are welcome and often necessary, the context in which the case study approach is applied is crucial. As Flyvbjerg (35) notes, in reference to the work of Thomas Kuhn, “a discipline without a large number of thoroughly executed case studies is a discipline without systematic production of exemplars, and that a discipline without exemplars is an ineffective one” (2006, P.27). Here, we present the Green Club programme as a case study for facilitating climate action through a sporting organisation acting as a regime-based grassroots intermediary, applying a previous application (36). Three specific projects are presented to create the overall outline of the Green Club programme. The aim is not to develop new conceptual models, or compare experiential dimensions of programme implementation, but rather to add a contribution to a body of literature on climate action through sport which is emerging, as indicated across this special issue.

The primary focus of the research approach was document analysis, following Bowen's (37) three phase approach: 1) skimming documents to grasp overall content, 2) identifying relevant analytic categories, and 3) interpreting the body of documents. Presented below are the results from the document analysis phase of the research approach. This approach helped to enable the contextual understanding of the Green Club programme as an organisational phenomenon within the GAA. In the results, we outline projects related to biodiversity, energy, and water, which have been prepared through document analysis and as such represent projects as they were self-reported. The cases used were selected as reporting documentation was provided. In the categories of Waste and Travel only video testimonies were provided as materials and have therefore been excluded from the document analysis.

## Results

Through undertaking an intrinsic case study approach, we have used document analysis to firstly situate the emergence of the Green Club programme before outlining three examples of local community action within Green Clubs, focussing on the strands of biodiversity, energy, and water. The information used is summarised as documented through the official reporting channels available publicly.

### The implementation of the green club programme

The GAA Green Club Programme was established in response to increasing demand from Gaelic Athletic Association (GAA) clubs and facilities for leadership and guidance in advancing sustainability and climate action. This emergent need was documented through the development of a survey in 2020, whereby over 200 clubs responded to reflect upon the positioning of sustainability in relation to their clubs’ actions and remit. The Green Club programme is currently in its third phase of implementation. For the purposes of the research presented here, we will focus on the first 2 phases which have now been finalised. In Phase 1, 45 clubs were represented through the process. Approximately 200 Clubs were formally involved in Phase 2 of the Programme representing all 32 counties across the country. To support clubs, the GAA Green Club Toolkit was developed to assist in adopting practical and effective sustainability initiatives. Organised across the five thematic areas (Energy, Water, Waste, Biodiversity, and Travel), the Toolkit offers tailored guidance and actionable recommendations specific to the operational context of GAA clubs. Each section includes case studies highlighting successful sustainability practices implemented by clubs across Ireland.

In addition to the thematic toolkits, supplementary overview documents were provided to facilitate navigation and application of the resources. These documents outline the contents and usage of the Toolkit, identify available local supports, suggest potential funding sources, and acknowledge the contributions of expert partners involved in the Toolkit's development. The Toolkit is also aligned with the Sustainable Development Goals (SDGs), reflecting the GAA Green Club Programme's broader commitment to environmental responsibility. While all Clubs could access and utilise the toolkit, these Clubs were supported through a series of milestones to be recognised as official GAA Green Clubs. Phase 2 ran through 2023 and 2024. Phase 3: Phase 3 is now open and will run from March 2025 to November 2026. We outline three case studies of projects developed through the Green Club programme across the five thematic areas.

#### Biodiversity: Mullingar Shamrocks, Co. Westmeath

As part of the GAA Green Club Programme (Phase 1), Mullingar Shamrocks GAA Club launched a thorough biodiversity initiative and became a mentor club for other clubs concentrating on biodiversity enhancement. Before this, in 2019, the club entered the Tidy Towns Special Award for Sustainable Development to begin its biodiversity efforts. Along an 860-meter biodiversity walking path, it erected 17 explanatory signs as part of this submission. In addition to designating their grounds as pesticide and herbicide free and encouraging organic weed control techniques like vinegar and salt sprays in place of traditional herbicides, Mullingar Shamrocks built bug hotels and bird and bat houses to help the local fauna.

The club created two separate wildflower garden areas to further improve the grounds. One on a clay embankment with flowering weeds that draw butterflies and bees. The other, a space with native plants including birch, alder, crab-apple, mountain ash, and hazel, to promote insect biodiversity. In addition to these ecological improvements, the club installed a barefoot walking trail and sensory garden. Toadstools, colourful mushrooms, and painted tractor tires hosting pollinator-friendly flora are all part of the sensory area. Additionally, stimulating the senses and promoting tactile engagement with nature is a barefoot path with bamboo, mulch, stones, and fragrant plants (such as lavender and lemon balm). Additionally, Mullingar Shamrocks incorporated artistic elements and active community involvement. Working with a local tattoo artist, a painting with a biodiversity theme was completed over a concrete wall. Through workshops on biodiversity awareness, plants, and interpretive signage, partnerships were extended to nearby schools, community organisations, and active retiree groups. The club's outreach to community and educational audiences was increased by these measures. To promote awareness of biodiversity, schoolchildren, local employees, and retirees took part in guided hikes, treks, and other activities.

These initiatives were greatly aided by the support of outside partners, such as Westmeath County Council, LAWPRO, Mullingar Tidy Towns, HSE, and local funders. Through their partnership the project was sustained thanks to grants, knowledge, and useful tools. In conclusion, the biodiversity program of the Mullingar Shamrocks GAA Club is an example of a multifaceted strategy for improving ecological knowledge and habitat quality in a community sports environment. The club approached grassroots biodiversity management as a process to be incorporated into sports infrastructure through habitat construction, organic maintenance techniques, sensory engagement, innovative community outreach, and solid local collaborations.

#### Energy: Connacht GAA Centre of Excellence

Since its opening in 2012, the Connacht GAA Centre of Excellence (CoE) in Bekan, County Mayo, has focused on incorporating the values of social, economic, and environmental sustainability. From initial planning to long-term administration, the stated goal was to connect these three sustainability pillars so that the Centre could meet innovation benchmarks and community requirements without sacrificing environmental integrity. The CoE's 1,200 square metre main structure, which covers 85 acres, has meeting rooms, a healthcare centre, offices, a canteen and changing facilities. There are five grass fields, one artificial turf field, a running track, a gym, a portable stand, and an indoor facility, which has a full-size indoor field. The complex has a 2.5 km floodlit walking trail around it.

The project was supported by a Sustainability Roadmap, which made energy management a priority concern. A lower carbon footprint, lower operating expenses, and a welcoming and comfortable environment all year long were the goals of this strategic focus. The Centre's Biodiversity and Sustainability Committee was founded on the basis of regular energy and sustainability briefings. Energy efficiency in building design, a building management system that monitors energy usage in real time and displays data throughout the facility, the creation of a SEAI Sustainable Energy Community (SEC), the installation of two 150 kW solar PV arrays, the transition to LED lighting for both indoor and outdoor areas that is integrated with sensors, timers and photocells, and two 22 kW EV charging stations that are powered by solar electricity. These interventions resulted in notable energy savings—both from real-time monitoring and LED upgrades—and ensured that much of the electricity demand was met through renewables. Revenue is generated from surplus solar energy exported to the grid.

In terms of community and environmental impact, the floodlit walkway offers an alternative to road walking during dark months for locals. Extensive biodiversity initiatives have involved planting 25,000 native trees (17,000 planted post-2021), hosting beehives, creating habitats for pollinators, and planting wildflowers and shrubs along the walking paths. These projects enhanced ecological value and engaged local volunteers and schools, of particular social value during periods when indoor gatherings were restricted during the coronavirus pandemic. The successes at Bekan inspired broader action across Connacht. Clubs in Mayo established a county-wide Sustainable Energy Community, while Castlebar Mitchels and the Mayo County Board launched a climate strategy for MacHale Park. A series of sustainability workshops, supported by the CoE, were delivered to local clubs and county boards. This model was expanded across Connacht in 2023. Some Key factors underpinning the CoE's success included the continuous measurement and monitoring of energy and water usage to identify efficiency opportunities, the adoption of an incremental approach by beginning with simple actions and scaling toward more ambitious sustainability projects, and strong partnerships with organizations such as SEAI, ATU Sligo, independent engineering firms, regional bodies, local authorities, Teagasc, schools, and the Regional Waste Management Offices. An Energy Master Plan (launched November 2022) outlined a ten-year strategy, including plans for heat pumps, additional LED and wind projects, and upgrades to external floodlighting, alongside waste management and continued biodiversity efforts. Despite significant progress, challenges remain such as the decarbonization of the site's heating systems, which currently rely on natural gas.

#### Water: Affane-Cappoquin GAA & Camogie club

The Affane-Cappoquin GAA & Camogie Club is located below the high tide line and next to two rivers and is in a region which is open to environmental vulnerability, particularly flooding. Examining water use, waterway interaction, and the club's operations’ overall environmental impact was spurred by participation in the Green Club programme. Additionally, the program encouraged cooperation between the local Camogie club and the GAA on a joint environmental project. With assistance from the Local Authority Waters Programme (LAWPro) Community Officer, a core Water Group spearheaded the initiative. Together, they evaluated current water practices, developed leakage and usage reduction strategies, and raised knowledge of ecosystem health. Planning was further informed by online water maps.

One of the main technical interventions was the installation of water-saving equipment in the dressing rooms, such as automated tap, shower, and cistern shut-offs, to reduce wasteful use. To precisely monitor use and identify leaks, the county council provided a water meter. To catch rainwater from rooftop runoff, a water butt was also erected. Two public drinking water taps were also set up in the club to encourage sustainable water use. Initiatives to promote biodiversity and engage the public were also developed. On the pedestal of an old scoreboard, a moss mural was put up to promote water conservation. The club planted a range of native trees, repurposed benches, and erected awareness signs along a walkway to improve the ecological value and tourist experience. LAWPro led local schoolchildren on a water awareness walk.

By comparing usage to billing, the water meter allowed for real savings and increased responsibility. Club members and guests’ knowledge of environmental issues was raised by the painting, signs, and tree planting. The community's relationship to the local water history and ecosystems was strengthened by the educational outreach. The club's approach, with straightforward steps setting the groundwork for future advancement, was one of the factors contributing to the project's success. Strategic direction and creative effect were guaranteed by the leadership of a committed internal team, professional advice from LAWPro, and creative contributions. Implementation was further aided by outreach to nearby schools and contributions from club volunteers.

## Discussion

While further work to understand the implementation challenges of such work are needed, for the purposes of this paper, the three examples help to indicate the potential for the initiative to facilitate bottom-up action for sustainability and climate action. We must acknowledge the limitation whereby potential biases of the available data are noted. However, the results presented help to highlight the novel potential of nationally coordinated sports clubs initiatives, implemented at the local level. This research does not concern demonstrable behaviour change or emissions reduction but rather highlights a mechanism for national policy to be translated locally to action. At a high level, through outlining successful local projects across three of the five thematic areas we can see the legitimisation of sustainability work within clubs, beyond their primary sporting remit. These local projects show the potential for institutional alignment through the structuring of a programmatic approach to stimulating local climate action through established organisation and provides counter evidence to pursuing the establishment of sustainability specific local “hubs”.

### The GAA as a regime-based transition intermediary

Within the transition literature, intermediaries are seen to be key actors who can operate as meso-institutions between the macro and micro. Macro-institutions set public policies and regulatory frames (e.g., Governments policy, EU frameworks etc.) and micro-institutions work to comply to the rules and norms set out at the regulatory level. Meso-institutions then, are seen to bridge these two levels, to support the implementation of the regime level policies at the micro-level. They help to monitor, enforce, and support through feedback micro-level activities (38). As such, regime-based transition intermediaries are those who can carry out particular orders from the current regime or who appear when as regime actors they shift their focus to intermediary work. They are seen as actors who are working to bring about change within a dominant system. As seen previously, such roles emerge for organisations in taking on a new role (39, 40) beyond their primary remit (41). In the case of the GAA further nuance is added here as for both the clubs involved and the centralised organisation, this is a new area to explore in relation to sustainability, as similarly developed through their Healthy Clubs initiative (15).

Through the Green Club programme, the GAA has taken on a role as centrally coordinating local activity in alignment with national policy in relation to climate action, decarbonisation, biodiversity, and sustainability, acting as a regime-based transition intermediary. This is done through the translation of national climate goals into locally relevant actions which can be implemented at club level such as pollinator corridors (linked to national biodiversity objectives), or LED lighting and energy retrofitting (linked to national decarbonisation objectives). There is a brokering role played between communities and organisations (such as SEAI, LawPRO, and local authorities in the local projects outlined). The legitimation of national objectives through local community actions is supported through the framing of sustainability as core to the values of the GAA at a national level. The provision of both a toolkit and a phased approach makes the barrier to entry low-risk while capacity can be developed in a sequenced manner to build towards larger projects in future phases. In relation to regime-based transition intermediaries within the sports realm, further work is needed to gain a greater understanding of the exchanges which occur when both the local clubs and central governing authority move into a new area beyond a sporting remit.

### The ecological civilising process

The historical importance of the GAA is well documented with regards to identity building and community formation for the relatively recent state of Ireland (13, 42). The civilising process as applied through sport through the work of Dunning and Elias and has been applied specifically to the Irish context in relation to hurling and Gaelic football (3, 4). Due to the deeply embedded nature of the GAA to local communities in the Irish context we here suggest a further “civilising”- for want of a better word- may be possible in relation to an environmental ethos. Within environmental sociology, the ‘ecological civilising process’ has been conceptually developed as it relates to the long-term development of environmental norms as part of a societal transition [see: (43, 44)]. Throughout its history the GAA has played a role in shaping Irish life. Through the Green Club programme, it provides an insight into the means through which climate action can be facilitated by regime-based transition intermediaries, to generate greater environmental behaviours and practices.

In the projects presented here we see the diversity of formations which clubs take. The flexibility of the programme across the different thematic areas, allows for locally specific projects to emerge, building on embedded strengths. Through such flexibility, the potential to embed environmental practices from a starting point towards the wider ‘diffusion of sustainability’ (45) is possible. We see energy monitoring, water metering, and new biodiversity practices emerge across our local project outlines bringing forward new behaviours. Coupled with this, they take an inter-generational approach, engaging different age groups from children to retirees. Also, across the cases we see the use of visible interventions from walking trails to signage and murals which support the normalisation of environmental sustainability with the club and wider community, moving beyond activism, campaigns, or specific programmes.

### Climate clubs or the co-opting of competition?

While the need for collaboration at the global, national, and local level is necessary to achieve climate action, the potential of competition also needs to be adequately addressed in relation to suggested mechanisms to facilitate participation at the local level. Previous research in relation to energy efficiency competitions (46) has shown that such approaches can massively scale up interventions. Competition alone is not sufficient for success and dependent on the area of focus, wider supports and capacity building is needed, such as through dedicated service provision, access to funding, and educational outreach amongst other factors. In the case of energy efficiency, it was shown as being a cost-effective measure. For the GAA and Green Clubs, the programme is not a competition but instead may act upon the inherent or embedded nature of competition generated between different local clubs across generations. This is symbolic of reputational competition is in-keeping with the “we-group” identities embedded in local GAA clubs (4). A potential development in relation to such a programme could seek to lean more heavily on aspects of this competitive spirit for which sports clubs are energised, as previously suggested through the development of prizes for activation of rural regions (47). Local communities have been shown to respond favourably to competitions to stimulate local developments more broadly, with focus given to their own local town or village even when wider cooperation is called for (48).

The creation of shared identity through sports clubs, acutely delivered considering the strategic nation building objectives as set out through the historical development of the GAA, provides fertile ground to enable collective climate action at the local level. Local sports clubs are underrepresented areas of study in climate research (49). Coupled with this, sport as a global industry is under increasing pressure regarding its environmental impact (50, 51). In the global context of the challenge of climate change, while potential benefits of community action are clear, there remains a danger that voluntary sports organisations are co-opted beyond their primary remit to undertake localised tasks in a vacuum of global scale action, leading to increased disillusionment, cynicism, and disconnection. This has previously been shown in relation to the enhancement of a local community competition to include sustainability topics, moving beyond the traditional scope (36). How to manage the expansion of sports club objectives to include grassroots climate action must be careful balanced to not overburden volunteers (52–54) and should seek instead to design such interventions from the perspective of local benefit above more abstract global change processes.

## Conclusion

Ireland has in recent years, innovated in relation to public engagement in climate action (55). This research paper situates the move towards sporting organisations as a mechanism for public and community engagement in climate action. As previously noted, “it is generally acknowledged that the formation of the GAA in 1884 was related to broader changes involving the rise of cultural nationalism in Ireland during the late nineteenth century. Yet more contemporary developments in both Gaelic games and in the organisational structure of the GAA tend not to be linked with wider social changes to the same extent” [(5), P. 2]. Here, we present an intrinsic case study of the Green Club programme as a foundational study, which can support further work- as has been seen with the Healthy Clubs programme (15)- to investigate empirically the value of the programme to facilitate wider social change over a long-term period.

Within the literature, the Green Club programme can be usefully compared with other initiatives internationally on the topic of enabling climate action through sports clubs at the collective level. In order to enable a whole of society approach to climate action, initiatives are needed which reach to all segments of society. Sports clubs represent potentially fertile ground to help build grassroots capacity for action. This, however, must be undertaken in union with top-down leadership in this space. Regime-based transition intermediaries can operate as key actors to translate policy to the local level, give legitimacy to national agendas, and support local capacity building on sustainability. Despite the unique characteristics of the GAA and its history, the emergence of regime-based intermediation of sustainability objectives may be explored through a range of different sport clubs (e.g. soccer, tennis etc.).

## Data Availability

Publicly available datasets were analyzed in this study. This data can be found here: www.gaa.ie.

## References

[1] Giulianotti R. Sport: A Critical Sociology. New Jersey: John Wiley & Sons (2015).

[2] Elias N Dunning E. The quest for excitement in leisure. Society and Leisure. (1986) 2:50–85.

[3] Dolan P Connolly J. The civilizing of hurling in Ireland. Sport in Society. (2009) 12(2):196–211. 10.1080/17430430802590995

[4] Connolly J Dolan P. The civilizing and sportization of gaelic football in Ireland: 1884–2009. J Hist Sociol. (2010) 23(4):570–98. 10.1111/j.1467-6443.2010.01384.x 21132949

[5] Connolly J Dolan P. Gaelic Games in Society. Cham: Springer International Publishing (2020).

[6] Bradley J. Gaelic games. In: Richard G Roland R, editors. Routledge Handbook of Global Sport. Oxfordshire: Routledge (2020). p. 481–9.

[7] Moynihan M. GAAconomics: The Secret Life of Money in the GAA. Dublin: Gill & Macmillan Ltd (2013).

[8] Jackman PC Lane A Wells N Kirby K Bird MD. The psychology of gaelic games: a co-produced scoping review to inform research, policy, and practice. Int J Sport Exerc Psychol. (2024) 22(9):2234–58. 10.1080/1612197X.2023.2257214

[9] Ryan L. The GAA:'Part of What We Are': A Centenary Assessment. Maynooth: The Furrow (1984). p. 752–64.

[10] Duffy M. ‘Skorts v Shorts: ‘They’re Awkward, and if it's your Time of the Month, it's just not Comfortable’’. Dublin: The Irish Times (2025. Available online at: [URL] (Accessed: [date of access]).

[11] Cronin M. Sport and a sense of irishness. Irish Stud Rev. (1994) 3(9):13–7. 10.1080/09670889408455460

[12] Rouse P. Sport and Ireland: A History. Oxfordshire: Oxford University Press (2017). [Google Scholar].

[13] Liston K. The GAA and the sporting Irish. In: Tom I, editor. Are the Irish Different? Manchester: Manchester University Press (2015). p. 199–210.

[14] Lane A Murphy N Regan C Callaghan D. Health promoting sports club in practice: a controlled evaluation of the GAA healthy club project. Int J Environ Res Public Health. (2021) 18(9):4786. 10.3390/ijerph18094786 33946150 PMC8124624

[15] Brennan MA Dolan P Regan C Alter T. Sport as a catalyst for social justice and inclusion: a case study of the gaelic athletic association's role in community and youth development. Youth. (2025) 5(3):70. 10.3390/youth5030070

[16] Avelino F Wittmayer JM. Shifting power relations in sustainability transitions: a multi-actor perspective. J Environ Policy Plan. (2016) 18(5):628–49. 10.1080/1523908X.2015.1112259

[17] Wittmayer JM Avelino F van Steenbergen F Loorbach D. Actor roles in transition: insights from sociological perspectives. Environ Innov Soc Transitions. (2017) 24:45–56. 10.1016/j.eist.2016.10.003

[18] Avelino F Wittmayer JM. The transformative potential of plural social enterprise: a multi-actor perspective. In: Jacques D Marthe N, editors. Theory of Social Enterprise and Pluralism. Oxfordshire: Routledge (2019). p. 193–221.

[19] Fischer LB Newig J. Importance of actors and agency in sustainability transitions: a systematic exploration of the literature. Sustainability. (2016) 8(5):476. 10.3390/su8050476

[20] Kivimaa P Boon W Hyysalo S Klerkx L. Towards a typology of intermediaries in sustainability transitions: a systematic review and a research agenda. Res Policy. (2019) 48(4):1062–75. 10.1016/j.respol.2018.10.006

[21] Bird C Barnes J. Scaling up community activism: the role of intermediaries in collective approaches to community energy. People Place Policy Online. (2014) 8(3):208–21. 10.3351/ppp.0008.0003.0006

[22] Kanda W Magnusson T Hjelm O. Intermediaries and Intermediation in Sustainability Transitions (2024).

[23] Moss T. Intermediaries and the governance of sociotechnical networks in transition. Environ Plan A. (2009) 41(6):1480–95. 10.1068/a4116

[24] Kern F Rogge KS. The pace of governed energy transitions: agency, international dynamics and the global Paris agreement accelerating decarbonisation processes? Energy Res Soc Sci. (2016) 22:13–7. 10.1016/j.erss.2016.08.016

[25] van Veelen B. Caught in the middle? Creating and contesting intermediary spaces in low-carbon transitions. Environ Plan C Politics Space. (2020) 38(1):116–33. 10.1177/2399654419856020

[26] Rocher L Verdeil É. Dynamics, tensions, resistance in solar energy development in Tunisia. Energy Res Soc Sci. (2019) 54:236–44. 10.1016/j.erss.2019.04.010

[27] Janda KB Reindl K Blumer Y Parag Y Wade F. Making more of middles: advancing the middle-out perspective in energy system transformation. Eceee Summer Study Proceedings (Vol. 2019). European Council for an Energy Efficient Economy (ECEEE) (2019). p. 199–204.

[28] Parag Y Janda KB. More than filler: middle actors and socio-technical change in the energy system from the “middle-out”. Energy Res Soc Sci. (2014) 3:102–12. 10.1016/j.erss.2014.07.011

[29] Matschoss K Heiskanen E. Making it experimental in several ways: the work of intermediaries in raising the ambition level in local climate initiatives. J Cleaner Prod. (2017) 169:85–93. 10.1016/j.jclepro.2017.03.037

[30] Klerkx L Leeuwis C. Establishment and embedding of innovation brokers at different innovation system levels: insights from the Dutch agricultural sector. Technol Forecast Soc Change. (2009) 76(6):849–60. 10.1016/j.techfore.2008.10.001

[31] Boon WP Moors EH Kuhlmann S Smits RE. Demand articulation in emerging technologies: intermediary user organisations as co-producers? Res Policy. (2011) 40(2):242–52. 10.1016/j.respol.2010.09.006

[32] Stewart J Hyysalo S. Intermediaries, users and social learning in technological innovation. Int J Innov Manag. (2008) 12(03):295–325. 10.1142/S1363919608002035

[33] Cronin M. ‘Is it for the glamour?’: masculinity, nationhood and amateurism in contemporary projections of the gaelic athletic association. In: Wanda B Anne M, editors. Irish Postmodernisms and Popular Culture. London: Palgrave Macmillan UK (2007). p. 39–51.

[34] Kavanagh D Cusack M. From performativity to normativity: the gaelic athletic association as a case in point. Cult Organ. (2021) 27(2):98–114. 10.1080/14759551.2020.1769621

[35] Flyvbjerg B. Five misunderstandings about case-study research. Qual Inq. (2006) 12(2):219–45. 10.1177/1077800405284363

[36] Boyle E Watson C Mullally G Gallachoir BO. Regime-based transition intermediaries at the grassroots for community energy initiatives. Energy Res Soc Sci. (2021) 74:101950. 10.1016/j.erss.2021.101950

[37] Bowen GA. Document analysis as a qualitative research method. Qual Res J. (2009) 9(2):27–40. 10.3316/QRJ0902027

[38] de Mello AM Schnaider PSB Saes MSM Souza-Piao R Nunes R Silva VL. Meso-institutions as systemic intermediaries in sustainable transitions governance. Environ Innov Soc Transitions. (2024) 52:100870. 10.1016/j.eist.2024.100870

[39] Martiskainen M Kivimaa P. Creating innovative zero carbon homes in the United Kingdom—intermediaries and champions in building projects. Environ Innov Soc Transitions. (2018) 26:15–31. 10.1016/j.eist.2017.08.002

[40] Elzen B Van Mierlo B Leeuwis C. Anchoring of innovations: assessing Dutch efforts to harvest energy from glasshouses. Environ Innov Soc Transitions. (2012) 5:1–18. 10.1016/j.eist.2012.10.006

[41] Kanda W Kuisma M Kivimaa P Hjelm O. Conceptualising the systemic activities of intermediaries in sustainability transitions. Environ Innov Soc Transitions. (2020) 36:449–65. 10.1016/j.eist.2020.01.002

[42] Keane J. The Role of the GAA in Creating, Maintaining and Reinforcing Senses of Place and Identity (Doctoral dissertation). Maynooth: National University of Ireland Maynooth (2001).

[43] Quilley S. The land ethic as an ecological civilizing process: Aldo Leopold, Norbert Elias, and environmental philosophy. Environ Ethics. (2009) 31(2):115. 10.5840/enviroethics200931215

[44] Rohloff A Saramago A. Climate Change, Moral Panics and Civilization. Oxfordshire: Routledge (2018).

[45] Boyle E McGookin C de Bhailís D Gallachóir BÓ Mullally G. The diffusion of sustainability and Dingle Peninsula 2030. UCL Open Environ. (2022) 4. 10.14324/111.444/ucloe.000 37228479 PMC10171406

[46] Vine EL Jones CM. Competition, carbon, and conservation: assessing the energy savings potential of energy efficiency competitions. Energy Res Soc Sci. (2016) 19:158–76. 10.1016/j.erss.2016.06.013

[47] Zeigermann U Kammerer M Böcher M. What drives local communities to engage in climate change mitigation activities? Examining the rural–urban divide. Rev Policy Res. (2023) 40(6):894–919. 10.1111/ropr.12528

[48] Nørgaard H Thuesen AA. Rural community development through competitions, prizes, and campaigns: the villagers’ perspective. J Rural Stud. (2021) 87:465–73. 10.1016/j.jrurstud.2020.03.006

[49] Wilby RL Orr M Depledge D Giulianotti R Havenith G Kenyon JA The impacts of sport emissions on climate: measurement, mitigation, and making a difference. Ann N Y Acad Sci. (2023) 1519(1):20–33. 10.1111/nyas.14925 36377356 PMC10098608

[50] Trendafilova S McCullough B Pfahl M Nguyen SN Casper J Picariello M. Environmental sustainability in sport: current state and future trends. Glo J Adv Pure Appl Sci. (2014) 3(1):9–14.

[51] Wicker P Thormann TF. Environmental impacts of major sport events. In: Milena MP Jean-Loup C, editors. Research Handbook on major Sporting Events. Cheltenham: Edward Elgar Publishing (2024). p. 373–85.

[52] Polk E. Momentum in the age of sustainability: building up and burning out in a transition town. In: Jenna C Caroline EL, editors. Perma/Culture. London: Routledge (2018). p. 97–105.

[53] Watson C Boyle E Mullally G Gallachóir BÓ. Responding to the Energy Transition in Ireland: The Experience and Capacity of Communities. Frankfurt, Germany: EPA (2020).

[54] Boyle E Ó Gallachóir B Mullally G. Participatory network mapping of an emergent social network for a regional transition to a low-carbon and just society on the dingle Peninsula. Local Environ. (2022) 27(12):1431–45. 10.1080/13549839.2021.1936472

[55] Wagner PM Lima V. The interests, ideas, and institutions shaping public participation in local climate change governance in Ireland. Local Environ. (2024) 29, 1170–84.

